# Association between BMI and oncologic outcomes in epithelial ovarian cancer: a predictors-matched case-control study

**DOI:** 10.1007/s00404-024-07537-8

**Published:** 2024-05-07

**Authors:** Gabriel Levin, Yoav Brezinov, Yossi Tzur, Tomer Bar-Noy, Melica Nourmoussavi Brodeur, Shannon Salvador, Susie Lau, Walter Gotlieb

**Affiliations:** 1 Division of Gynecologic Oncology, Jewish General Hospital, McGill University, Montreal, QC Canada; 2 Segal Cancer Center, Lady Davis Institute of Medical Research, McGill University, Montreal, QC Canada

**Keywords:** Case-control, Matched, Obesity, Ovarian cancer, Survival

## Abstract

**Objective:**

We aimed to study the association between obesity and survival in ovarian cancer (OC) patients, accounting for confounders as disease stage, histology, and comorbidities.

**Methods:**

Retrospective matched case-control study of consecutive patients, with epithelial OC. Obese (body mass index [BMI] ≥ 35 kg m^−2^) patients were matched in a 1:4 ratio with patients having lower BMIs (BMI < 35 kg m^−2^) based on disease stage, cytoreduction state, tumor histology and ASA score. We compared the 3-year and total recurrence-free survival and overall survival through Kaplan–Meier survival curves and Cox proportional hazards.

**Results:**

Overall, 153 consecutive patients were included, of whom 32 (20.9%) had a BMI ≥ 35. and 121 a BMI < 35. The median follow-up time was 39 months (interquartile range 18–67). Both study groups were similar in multiple prognostic factors, including American Society of Anesthesiologists physical status, completion of cytoreduction, histology and stage of disease (*p* = 0.981, *p* = 0.992, *p* = 0.740 and *p* = 0.984, respectively). Ninety-five (62.1%) patients underwent robotic surgery and conversion rate from robotic to laparotomy was similar in both groups 2 (6.3%) in obese group vs. 6 (5.0%) in lower BMI patients, *p* = 0.673. During the follow-up time, the rate of recurrence was similar in both groups; 21 (65.6%) in obese group vs. 68 (57.1%), *p* = 0.387 and the rate of death events was similar; 16 (50.0%) in obese group vs. 49 (40.5%), *p* = 0.333). The 3-year OS was higher in the obese group (log rank *p* = 0.042) but the 3-year RFS was similar in both groups (log rank *p* = 0.556). Median total OS was similar in both groups 62 months (95% confidence interval 25–98 months) in obese vs. 67 months (95% confidence interval 15–118) in the lower BMI group, log rank *p* = 0.822. Median RFS was similar in both groups; 61 months (95% confidence interval 47–74) in obese, vs. 54 (95% confidence interval 43–64), log rank *p* = 0.842. In Cox regression analysis for OS, including obesity, age, laparotomy and neoadjuvant treatment – only neoadjuvant treatment was independently associated with longer OS: odds ratio 1.82 (95% confidence interval 1.09–3.05) and longer RFS: odds ratio 2.16 (95% confidence interval 1.37–3.41).

**Conclusions:**

In the present study on consecutive cases of ovarian cancer, obesity did not seem to be associated with outcome, except for an apparent improved 3-year survival that faded away thereafter.

## What does this study add to the clinical work

**Table:** 

Obesity does not seem to be associated with ovarian cancer outcome, Except for an apparent improved 3-year survival that fades away thereafter.	

## Introduction

Ovarian cancer (OC) is the deadliest of gynecologic malignancies [1, 2], with an average lifetime risk of approximately 1.5%, [1, 3]. It is mostly diagnosed at a late stage and the stage at diagnosis is the most important predictor of overall survival [3]. Other main prognostic factors, among others, are age, tumor grade, and the amount of residual disease after cytoreduction [4].

In general, obesity is associated with greater mortality in patients with cancer [5]. However, in some specific cancers such as lung cancer, renal cell carcinoma, and melanoma obese patients have longer survival than their slimmer counterparts [5]. Several studies, including meta-analyses, have examined the association between obesity and OC survival, yielding conflicting results [6].

Considering the conflicting evidence, this study addresses the association of obesity with survival in OC patients taking into account confounders such as disease stage, histology, and comorbidities.

## Materials and methods

### Study population

The study population was composed of all consecutive patients who were diagnosed with and treated for International Federation of Gynecology and Obstetrics (FIGO) stages I–IV epithelial OC, including serous, endometrioid, clear cell and mucinous types, between 2006 and 2022. We categorized patient by body mass index (BMI) into two groups: (1) obese (BMI ≥ 35 kg m^−2^) and (2) lower BMI (BMI < 35 kg m^−2^).

### Study design

We matched cases (obese) to controls (lower BMI) in a 1:4 ration, matched by disease stage, cytoreduction status at end of surgery, American Society of Anesthesiologists (ASA) score and tumor histology. No tolerance for matching was allowed. Cases with no matching controls—were excluded (*n* = 8). Data for the study were collected retrospectively from a prospectively maintained computerized database. We extracted information for each patient, including age, BMI, histologic type, grade, International Federation of Gynecology and Obstetrics (FIGO) stage, extent of cytoreduction at surgery and residual disease, administration of neoadjuvant chemotherapy (NACT), and type of surgery (robotic vs. laparotomy vs. conversion from robotics to laparotomy). The extent of cytoreduction was defined as R0 if no macroscopic disease was observed at the end of the operation; R1 cytoreduction was defined as total disease measuring less than 1 cm, and R2 was defined as total disease measuring more than 1 cm.

Overall survival (OS) was defined as the time from diagnosis to either last follow-up or death. In patients who had recurrence-progression-free survival (PFS) was defined as the time from diagnosis to recurrence. Recurrences were diagnosed clinically by patient symptoms or radiologically when triggered by abnormal CA-125 levels.

## Statistical analysis

Statistical analysis was performed using SPSS 29 (IBM, Chicago, IL). Matching of cases to controls was performed by SPSS. Statistical significance was calculated using the chi-square test and Fischer’s exact test for differences in categorical variables, and the Mann–Whitney U test for continuous variables. Kaplan–Meier survival curves were used to calculate survival estimates (PFS and OS), and the log-rank test was used to quantify survival differences according to different variables. Cox regression analysis was performed to determine independent factors associated with OS and PFS.

### Ethics approval

An institutional review board approval (protocol #15–070) was granted for this study, with yearly reviews.

## Results

**Fig. 1:**
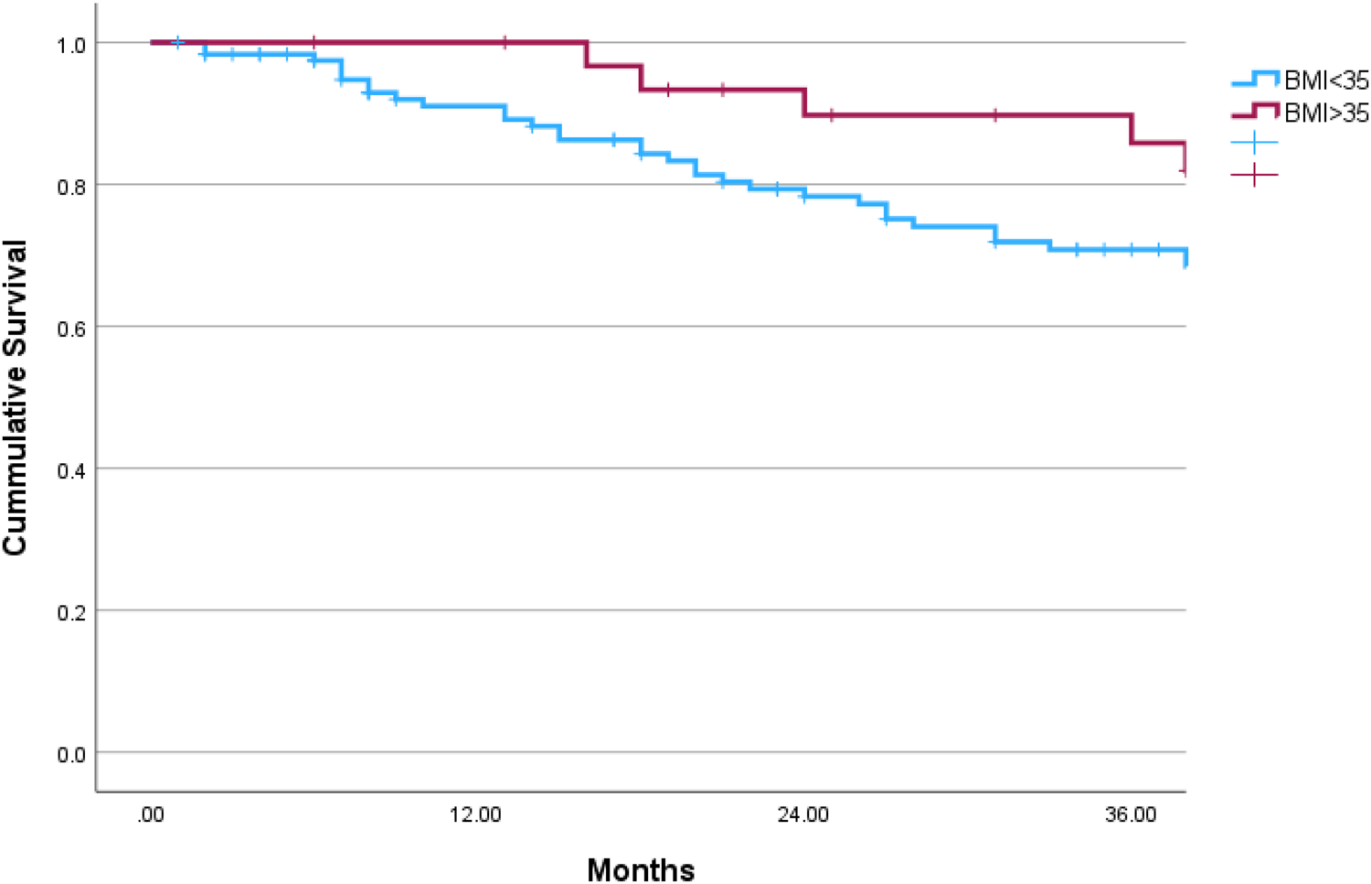
3-year overall survival of obese vs. lower BMI OC patients

**Fig. 2:**
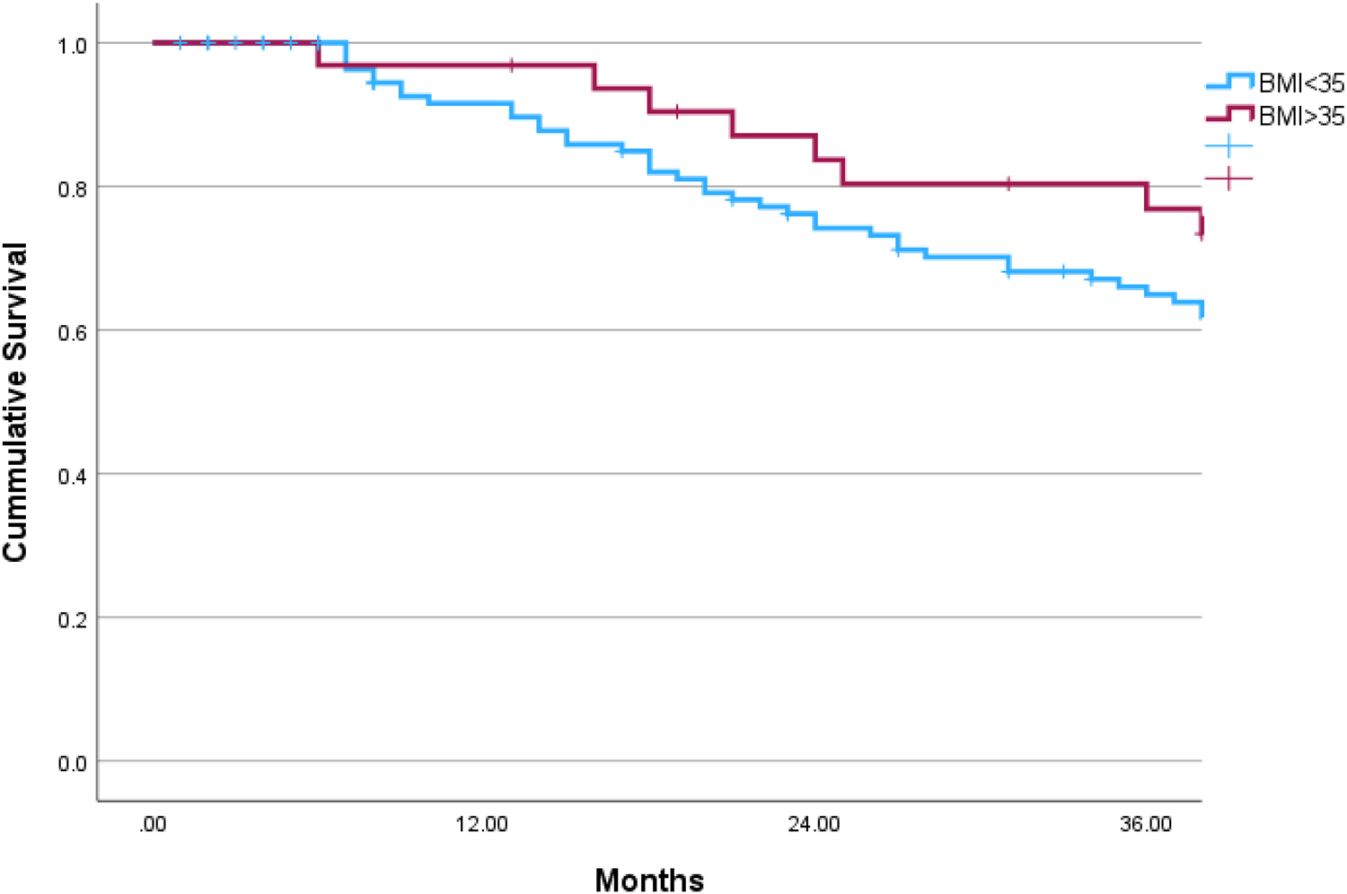
3-year recurrence-free survival of obese vs. lower BMI OC patients

**Fig. 3:**
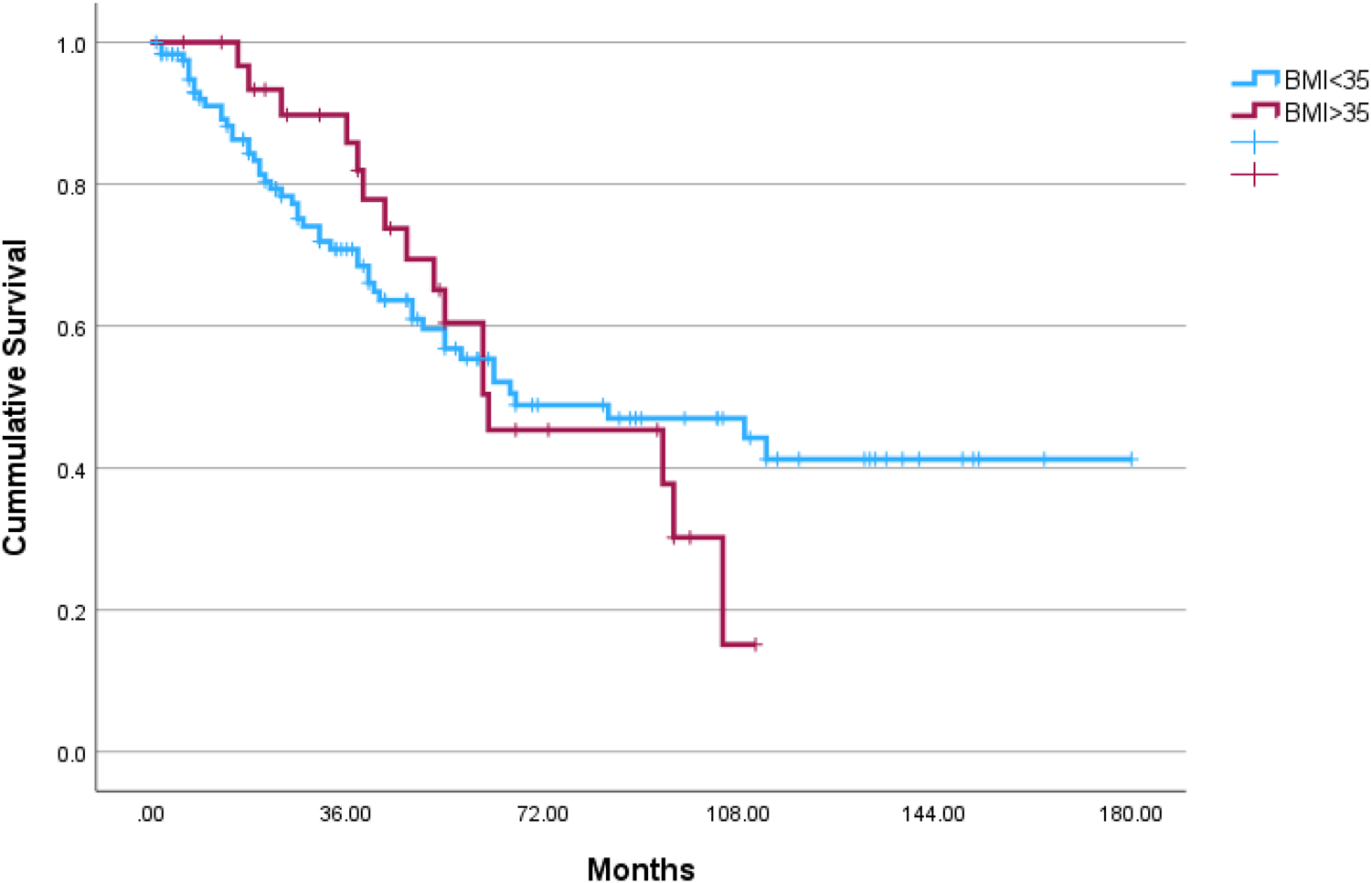
Total overall survival of obese vs. lower BMI OC patients

Overall, 153 patients were included in this study during 2006–2022, 32 (20.9%) obese patients (BMI ≥ 35.0) were matched with 121 patients with lower BMI. The median follow-up time was 39 months [interquartile range 18–67] and was similar in both groups (*p* = 0.572) (Table 1). The median BMI of the study groups were as follows: Obese 38.0 [IQR 36.0–41.9], lower BMI 24.6 [IQR 21.2–27.8]. Both study groups had similar ASA, residual disease after cytoreduction, histology and stage of disease (*p* = 0.981, *p* = 0.992, *p* = 0.740 and *p* = 0.984, respectively). Ninety-five (62.1%) patients underwent robotic surgery and conversion rate from robotic to laparotomy was similar in both groups 2 (6.3%) in obese group vs. 6 (5.0%) in lower BMI patients, *p* = 0.673. During the follow-up time, the rate of recurrence was similar in both groups; 65.6% (21 patients) in the obese group vs. 57.1% (68 patients), *p* = 0.387 and the rate of death events was similar; 50.0% (16 patients) in the obese group vs. 40.5% (49 patients), *p* = 0.333. Figure 1 presents 3-year OS in both study groups. The 3-year OS was higher in the obese group (log rank *p* = 0.042). Figure 2 present 3-year RFS, which was similar in both group (log rank *p* = 0.556). Survival curves for total OS and RFS are presented in Figs. 3and4. Median total OS was similar in both groups 62 months (95% confidence interval 25–98 months) in obese vs. 67 months (95% confidence interval 15–118) in lower BMI group, log rank *p* = 0.822. Median RFS was similar in both groups; 61 months (95% confidence interval 47–74) in obese vs. 54, 95% (confidence interval 43–64), log rank *p* = 0.842. Table 2 presents Cox regression for total OS, including obesity, age, laparotomy, and neoadjuvant treatment. Neoadjuvant treatment was the only independent factor associated with longer OS: odds ratio 1.82 (95% confidence interval 1.09–3.05). Table 3 presents Cox regression for RFS. Once again, neoadjuvant treatment was the only independent factor associated with longer RFS: odds ratio 2.16 (95% confidence interval 1.37–3.41).

**Table 1:** Comparison of obese vs. lower BMI group

Characteristics	Obese *n* = 32	Lower BMI *n* = 121	*p* value
Age, years	64 (55–73)	66 (57–75)	.340
Body mass index	38.0 (36.0–41.9)	24.6 (21.2–27.8)	< .001
ASA		64 (52.9%)	.981
2	17 (53.1%)	57 (47.1%)	
3	15 (46.9%)		
Follow-up, months	49 (26–71)	37 (15–67)	.572
Year of surgery	2014 (2010–2018)	2014 (2011–2019)	.743
Surgery mode			.239
Robotic	8 (25.0%)	50 (41.3%)	
Laparotomy	22 (68.8%)	65 (53.7%)	
Conversion of robotic to laparotomy	2 (6.3%)	6 (5.0%)	
Surgery type			.241
Primary cytoreduction	18 (56.3%)	54 (44.6%)	
Interval cytoreduction	14 (43.8%)	67 (55.4%)	
Cytoreduction			.992
R0	21 (65.6%)	78 (64.5%)	
R1	8 (25.0%)	31 (25.6%)	
R2	3 (9.4%)	12 (9.9%)	
Histology			.740
High-grade serous	26 (81.3%)	104 (86.0%)	
Endometroid	4 (12.5%)	12 (9.9%)	
Clear cell	1 (3.1%)	4 (3.3%)	
Mucinous	1 (3.1%)	1 (0.8%)	
Stage			.984
I	4 (12.5%)	17 (14.0%)	
II	5 (15.6%)	16 (13.2%)	
III	20 (62.5%)	76 (62.8%)	
IV	3 (9.4%)	12 (9.9%)	
Recurrence events	21 (65.6%)	68 (57.1%)	.387
Death events	16 (50.0%)	49 (40.5%)	.333

*ASA *American Society of Anesthesiologists

**Fig. 4:**
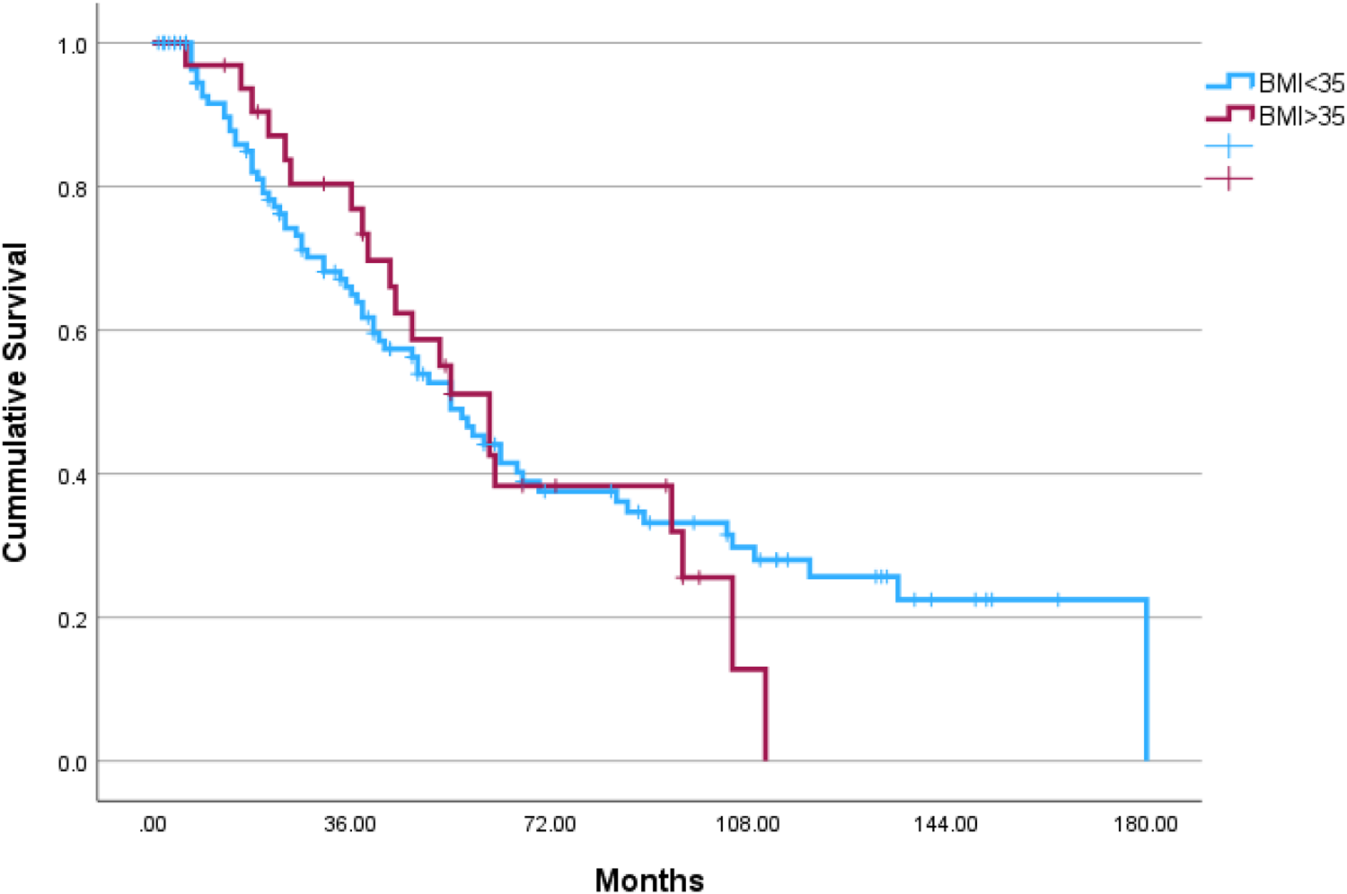
Total recurrence-free survival of obese vs. lower BMI OC patients

**Table 2:** Cox regression for total overall survival

Characteristic	*B*	Sig	OR	95.0% CI for OR	
				Lower	Upper
Obese	0.274	0.352	1.316	0.738	2.346
Age	0.021	0.082	1.021	0.997	1.046
Laparotomy	− 0.400	0.113	0.670	0.409	1.099
NACT	0.603	0.021	1.828	1.095	3.051

*NACT *neoadjuvant chemotherapy, *OR *odds ratio

**Table 3:** Cox regression for recurrence-free survival

Characteristic	*B*	Sig	OR	95.0% CI for OR	
				Lower	Upper
Obese	0.223	0.386	1.250	0.755	2.068
Age	0.018	0.088	1.018	0.997	1.039
Laparotomy	− 0.057	0.795	0.944	0.614	1.453
NACT	0.774	< 0.001	2.169	1.377	3.417

*NACT* neoadjuvant chemotherapy, *OR* odds ratio

## Discussion

The major finding from this study is that obese women (BMI ≥ 35 kg m^−2^) have similar OS to their slimmer counterpart. There is an advantage in 3-year OS for obese patients, probably driven by lower recurrence rates during this period, and OS curves meet back at 5-years of follow-up. The only independent factor associated with OS and RFS is treatment with neoadjuvant chemotherapy.

Obesity has emerged as a significant factor influencing survival outcomes in many malignancies [7]. As it is theoretically modifiable, it is drawing increasing attention from the medical community. While the prevalence of obesity continues to rise, understanding and addressing the impact of obesity on OC survival is becoming increasingly crucial, with potential implications for clinical management and public health strategies. In OC, research has inconsistently shown different results. Some studies support that obese individuals with OC face a higher risk of both diagnosis at advanced stages and poorer OS [8–11]. These studies raise several mechanisms that possibly underlie this association, including chronic inflammation, hormonal imbalances, and insulin resistance, which can facilitate the growth and spread of OC. Furthermore, obesity can complicate surgical and chemotherapy interventions, potentially leading to suboptimal treatment outcomes. However, other studies found contrasting results [12–15], suggesting the*‘obesity paradox’* [16]*.* Furthermore, many studies have found no association of BMI with OC survival [17–19].

Importantly, many of these studies did not account for prognostic factors other than BMI, which may explain these conflicting findings. It is therefore, that our study, which matched patients for comorbidities (e.g., ASA), disease stage, level of cytoreduction and the type of OC — sheds some additional light on the apparent controversy.

In our study, the 3-year survival rate was superior in obese patients. The recurrence-free survival in the first 3-years did not reach statistical significance, however the curves do separate. It is suggested that the superior 3-year OS may be driven by lower recurrence among obese patients in this period. One explanation may be that chemotherapy doses are calculated by weight formulas, and obese patients receive higher doses if not reduced as per patient’s tolerance [20]. However, it is possible that this gap may be explained by the obesity paradox.

The concept of the “obesity paradox” in cancer highlightens the unexpected phenomenon where obese individuals exhibit better outcomes in certain cancer types. While obesity is a well-established risk factor for various malignancies, recent studies have suggested that obese patients with cancer, particularly those with advanced stages or undergoing aggressive treatments, might experience improved survival rates. This intriguing observation has prompted extensive research into potential mechanisms underlying this paradox, such as enhanced energy reserves, metabolic adaptations, and differences in tumor biology [21–23].

Our study results indicating that obesity has no independent association with overall survival is in line with some previous literature [17, 18], however others have showed adverse outcomes [24], although none of these studies accounted for confounders as in our study.

Interestingly, the use of neoadjuvant chemotherapy was the only independent factor associated with improved survival in our study. While there are many confounders, this goes in line with a large study which underlined that higher provision of neoadjuvant chemotherapy is associated with improvements in median overall survival and larger decrease in the short-term mortality [25].

Our study has some limitations. First, this is a retrospective study, carrying inherent biases such as selection and information bias. Although in our study, all data were complete for each patient. Furthermore, as this is a retrospective study, it is impossible to account for many confounders, as provider bias, different treatment protocols as OC patients may undergo multiple lines of chemotherapy and dose interruptions. Furthermore, we must acknowledge the sample size of our study, which might be too small to identify statistical significance and to generalize. Further studies with larger sample size are needed to deepen the understanding of our findings. Nevertheless, the strength of our study is the matching in a 1:4 ratio for the most significant factors of disease outcome as disease stage and degree ofcytoreduction, as well as histology and patient’s comorbidities.

## Conclusions

In this study, we found a temporary association, with obese patients having a limited overall survival advantage for the first 3 years, that then fades away with no further association with oncological outcome.

## Data Availability

The data that support the findings of this study are not openly available due to reasons of sensitivity and are available from the corresponding author upon reasonable request.

## Abbreviations

OC: Ovarian cancer
OS: Overall survival,
RFS: Recurrence-free survival
